# A Systematic Review of Spaced Repetition, Retrieval Practice, and Academic Performance in Undergraduate Medical Students

**DOI:** 10.7759/cureus.116147

**Published:** 2026-09-12

**Authors:** Nathan K Luu, Scott Severance

**Affiliations:** 1 Education, Liberty University, Lynchburg, USA; 2 Medical Education, Liberty University College of Osteopathic Medicine, Lynchburg, USA

**Keywords:** active recall, anki, anki flashcards, spaced repetition, spaced repetition flashcards

## Abstract

Spaced repetition and active retrieval practice are learning strategies designed to improve long-term memory retention. These approaches target the forgetting curve, which describes the progressive loss of information over time unless learners actively recall and review material at spaced intervals. Modern learning platforms such as Anki and Quizlet use scheduled review and retrieval to strengthen memory consolidation and recall, and their use in undergraduate medical education has increased in recent years. This systematic review aimed to determine whether spaced repetition and active retrieval interventions improve memory retention and examination performance among undergraduate medical students.

The review followed PRISMA (Preferred Reporting Items for Systematic Reviews and Meta-Analyses) guidelines. PubMed, ERIC, and Scopus were searched for studies examining spaced repetition or retrieval practice in undergraduate medical students, and studies reporting objective academic outcomes were included. Of 314 records identified, 210 remained after duplicate removal, and 21 studies met the inclusion criteria. Due to heterogeneity in study designs, interventions, and outcome measures, a narrative synthesis was undertaken, and no meta-analysis was performed. Most controlled studies reported improvements in examination performance or knowledge retention following spaced retrieval interventions. Observational studies of self-directed flashcard use reported mixed findings. Several demonstrated positive associations with academic performance, whereas others, including studies that measured platform use objectively rather than by self-report, found no association.

These findings were more susceptible to self-selection bias and confounding, and most were judged to be at serious risk of bias. Several studies suggested greater benefit with sustained and longitudinal engagement. Overall, the available evidence suggests that spaced repetition and active retrieval practice may improve academic outcomes in undergraduate medical education, particularly when implemented longitudinally through structured retrieval. Controlled studies provided stronger support for these benefits, whereas evidence from observational studies of self-directed Anki use was weaker because of potential selection bias and confounding. Future research should focus on standardized implementation strategies and longer follow-up to determine whether early use of spaced retrieval produces durable benefits during clinical training and on future examinations.

## Introduction and background

Undergraduate medical education requires students to absorb and retain large volumes of information while preparing for summative examinations [1-3]. Spaced repetition and retrieval practice are related but distinct learning strategies. Spaced repetition involves reviewing information at intervals over time rather than during a single concentrated session, whereas retrieval practice requires learners to actively recall previously learned information through methods such as self-testing, flashcards, or practice questions [1-3]. Spaced repetition describes when material is reviewed, while retrieval practice describes how it is reviewed. Both strategies are informed by the forgetting curve, which illustrates the decline in recall that occurs when newly learned information is not reinforced [3]. Repeated retrieval at spaced intervals is intended to slow this decline and strengthen subsequent recall, as illustrated in Figures 1-2. Digital retrieval tools used by medical students include Anki and question banks; Anki combines active retrieval with algorithmically scheduled review [2,4-11]. Anki is a free, open-source flashcard application that adjusts the interval between reviews according to the learner’s reported difficulty in recalling each card. This process is illustrated in Figure 3. Evidence regarding spaced retrieval in undergraduate medical education includes observational studies of voluntary, self-directed Anki or question-bank use and controlled studies of faculty-developed flashcards, repeated quizzes, and curriculum-embedded retrieval activities [4-10,12-25].

**Figure 1:**
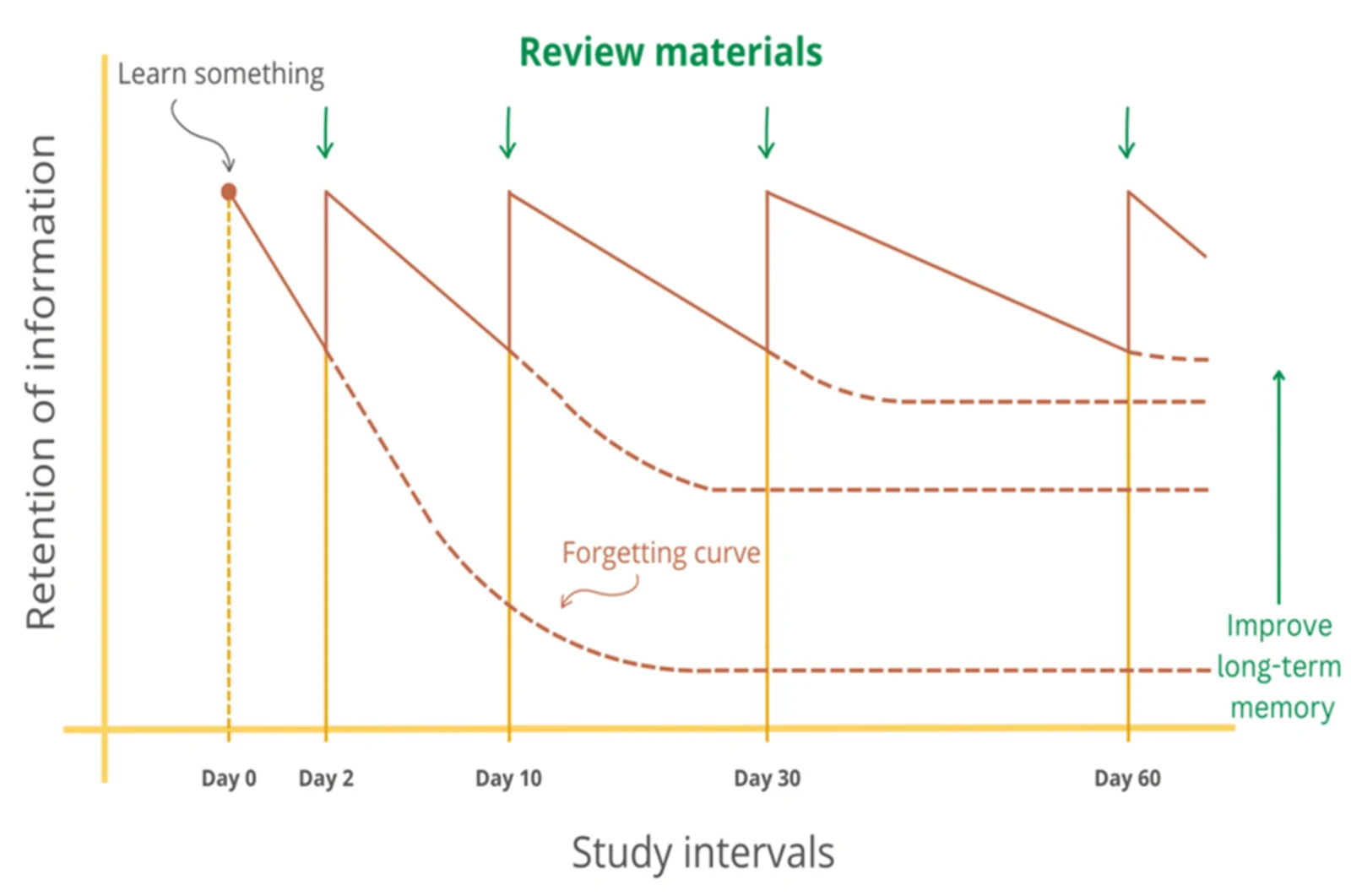
Illustration of the Ebbinghaus forgetting curve and the effect of spaced repetition on information retention Memory retention declines rapidly after initial learning, a pattern described by the forgetting curve (dashed line). In the absence of reinforcement, recall decreases over time. Periodic review sessions (solid vertical lines) halt this decline by restoring memory retention and reducing the rate of subsequent forgetting. When reviews are spaced at increasing intervals, each successful retrieval further reinforces memory and prolongs retention Reproduced from Wikimedia Commons. This file is made available under the Creative Commons CC0 1.0 Universal Public Domain Dedication. Available at: https://commons.wikimedia.org/wiki/File:Forgetting_curve_and_work_of_Ebbinghaus.png [26]

**Figure 2:**
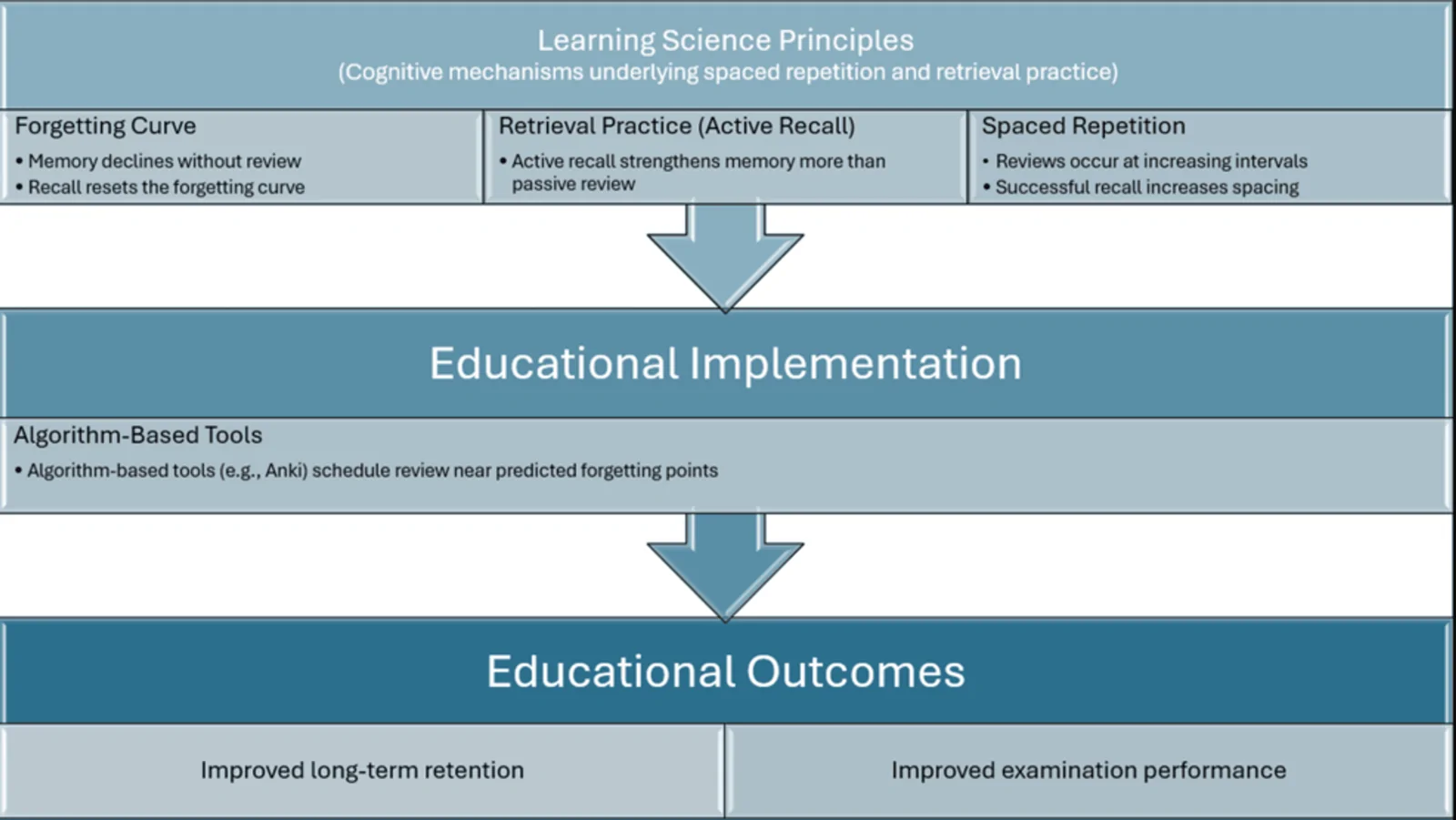
Principles of the science of learning This figure illustrates the relationship between core principles of learning and educational implementation. The top panel summarizes the forgetting curve, retrieval practice (active recall), and spaced repetition, which collectively all play a role in memory formation. Studies have shown that these principles are operationalized through algorithm-based tools (e.g., Anki, Quizlet) that schedule reviews at increasing intervals. The bottom panel highlights the associated educational outcomes, including improved long-term retention - defined as the ability to recall information over extended intervals (weeks to months) - and improved examination performance Image credits: Nathan Luu

**Figure 3:**
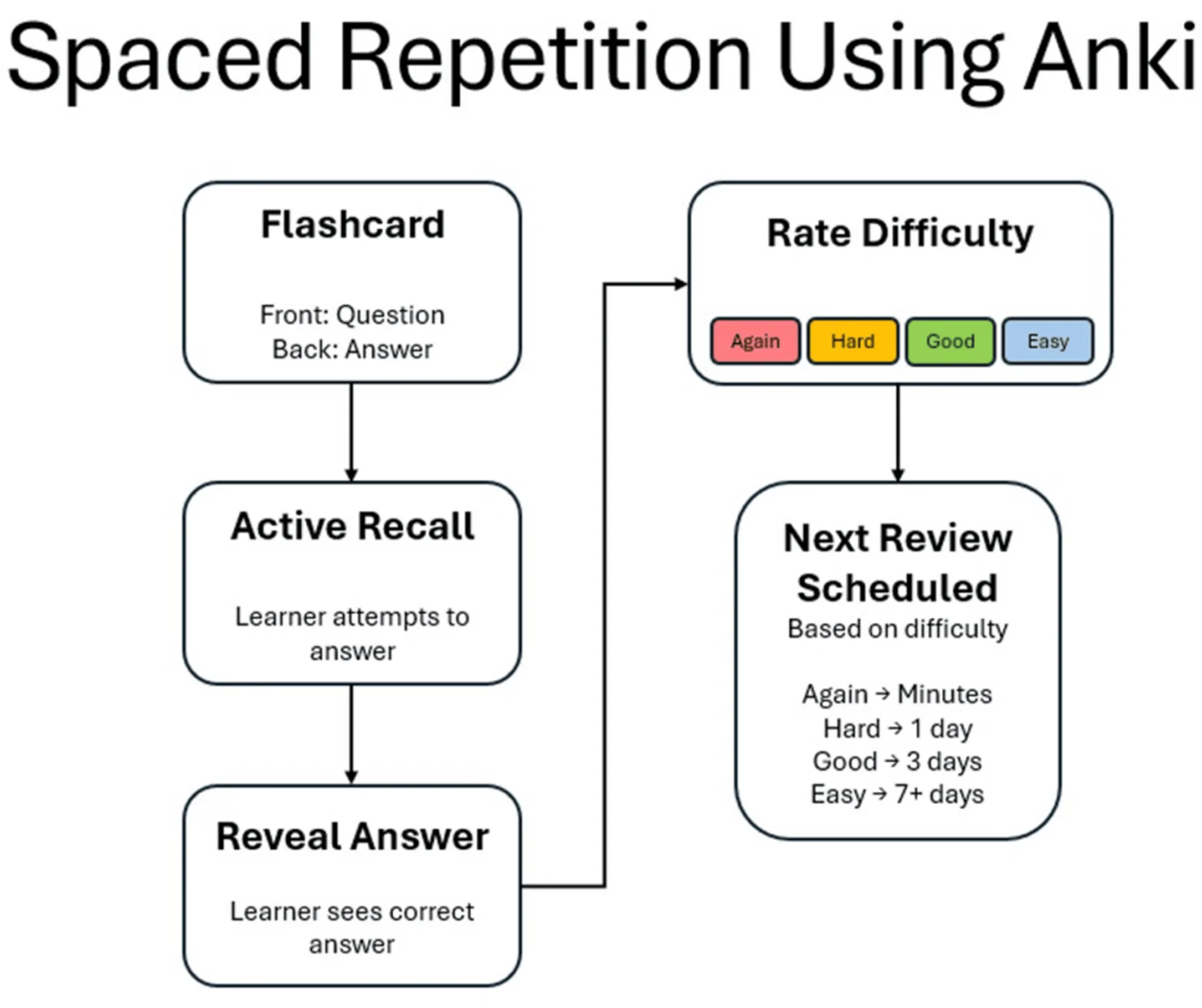
Mechanism of spaced repetition and active recall using Anki Anki is a digital flashcard platform intended to promote active recall and spaced repetition. Each card prompts the learner to retrieve an answer before revealing the correct response. After every card, the learner rates how difficult it was to recall the answer to the question on that card. This feedback determines when the card will be shown to the user again. Cards with answers that are recalled easily are reviewed less frequently and with longer periods of time between review sessions; cards testing information that was more difficult for the learner to recall are reviewed more frequently because the time interval between review sessions is shorter. Cards that are reviewed after intervals greater than 21 days are considered “mature.” Flashcards are organized into topic-based decks, allowing learners to easily select the topics covered in their review sessions. Consistent Anki use strengthens retention and reduces forgetting, thereby aiding long-term memory retention Image credits: Nathan Luu

These studies evaluate different outcomes. Course examinations, NBME (National Board of Medical Examiners) examinations, and licensing examinations measure academic performance at a particular point in time, whereas knowledge retention requires recall to be assessed after a defined interval. These outcomes are therefore not considered interchangeable.

Previous reviews have examined spaced repetition across undergraduate and postgraduate medical education, and one recent review specifically evaluated the relationship between Anki use and examination performance among undergraduate medical students [1,2,11]. However, the evidence remains heterogeneous across self-directed flashcard use and structured curricular retrieval interventions, particularly regarding the distinction between examination performance and delayed knowledge retention. Accordingly, this systematic review asked: Among undergraduate medical students, what evidence supports an association between spaced repetition and retrieval practice interventions and objective academic outcomes, specifically examination performance and delayed knowledge retention?

## Review

Methodology

This systematic review was conducted and reported in accordance with the Preferred Reporting Items for Systematic Reviews and Meta-Analyses 2020 (PRISMA 2020) guidelines. The review protocol was not prospectively registered. A completed PRISMA 2020 checklist is provided in the Appendices.

Eligibility Criteria

The inclusion and exclusion criteria used for study selection are summarized in Table 1. Studies were included if they focused on undergraduate medical students, examined spaced repetition or retrieval-based interventions, and reported measurable educational outcomes, whereas studies involving other learner populations or lacking objective outcomes were excluded.

**Table 1:** Inclusion and exclusion criteria This table summarizes the inclusion and exclusion criteria applied during title/abstract screening and full-text review. The criteria in the two columns are listed for summary purposes and are not intended to represent direct, row-by-row comparisons

Inclusion criteria	Exclusion criteria
Studies evaluating undergraduate medical students (preclinical or clinical years)	Studies involving postgraduate trainees or allied health students
Studies examining spaced repetition, distributed practice, or retrieval-based educational interventions	Studies not evaluating spaced repetition or retrieval-based strategies
Studies reporting an objective academic outcome, including course or block examination scores, post-intervention assessment scores, licensing or standardized examination scores, or delayed recall or retention assessments	Narrative reviews, commentary articles, or non-original research
Studies published in English	Studies reporting only learner perceptions, preferences, satisfaction, or platform usage without an objective academic outcome

Information Sources and Search Strategy

Electronic database searches were conducted on PubMed, ERIC, and Scopus on February 3, 2026. There were no date, publication-type, or other database filters. However, eligibility was limited to English-language studies. The reference lists of retrieved full-text articles were manually screened for additional eligible studies. Gray literature sources, including dissertations, conference abstracts, and trial registries, were not searched. The complete database-specific search strategies are provided in Table 2.

**Table 2:** Complete search strategy Search strategies used to identify studies examining spaced repetition, distributed practice, Anki, and retrieval practice in undergraduate medical education across PubMed, ERIC, and Scopus

Database	Search date	Search strategy	Database filters
PubMed	February 3, 2026	("spaced repetition" OR "distributed practice" OR Anki OR "retrieval practice") AND ("medical education" OR "medical student*" OR "undergraduate medical education")	None
ERIC	February 3, 2026	("spaced repetition" OR "distributed practice" OR Anki OR "retrieval practice") AND ("medical education" OR "medical student*" OR "undergraduate medical education")	None
Scopus	February 3, 2026	TITLE-ABS-KEY("spaced repetition" OR "distributed practice" OR Anki) AND TITLE-ABS-KEY("medical student*" OR "medical education")	None

Reviewer Workflow

Title and abstract screening, full-text eligibility assessment, data extraction, and risk-of-bias classification were primarily performed by one reviewer (NKL) under the supervision of the senior author (SS). These stages were not conducted independently in parallel. Questions regarding study eligibility, extracted information, or interpretation were discussed with the senior author and resolved through consensus. Both authors contributed to the narrative synthesis and manuscript revision.

Study Selection

The study selection process is summarized in Figure 4. A total of 314 records were identified through PubMed, ERIC, and Scopus. After 104 duplicate records were removed, 210 records underwent title and abstract screening, of which 150 were excluded. Sixty reports were sought for retrieval, and three could not be retrieved. Fifty-seven full-text articles were assessed for eligibility. Thirty-six articles were excluded based on the predefined eligibility criteria, and 21 studies were included in the final narrative synthesis. After full-text reassessment, Mishall et al. [27] and Laurent and Phillips [28] were excluded because they reported learner preference, perceived effectiveness, or platform usage without an objective academic outcome.

**Figure 4:**
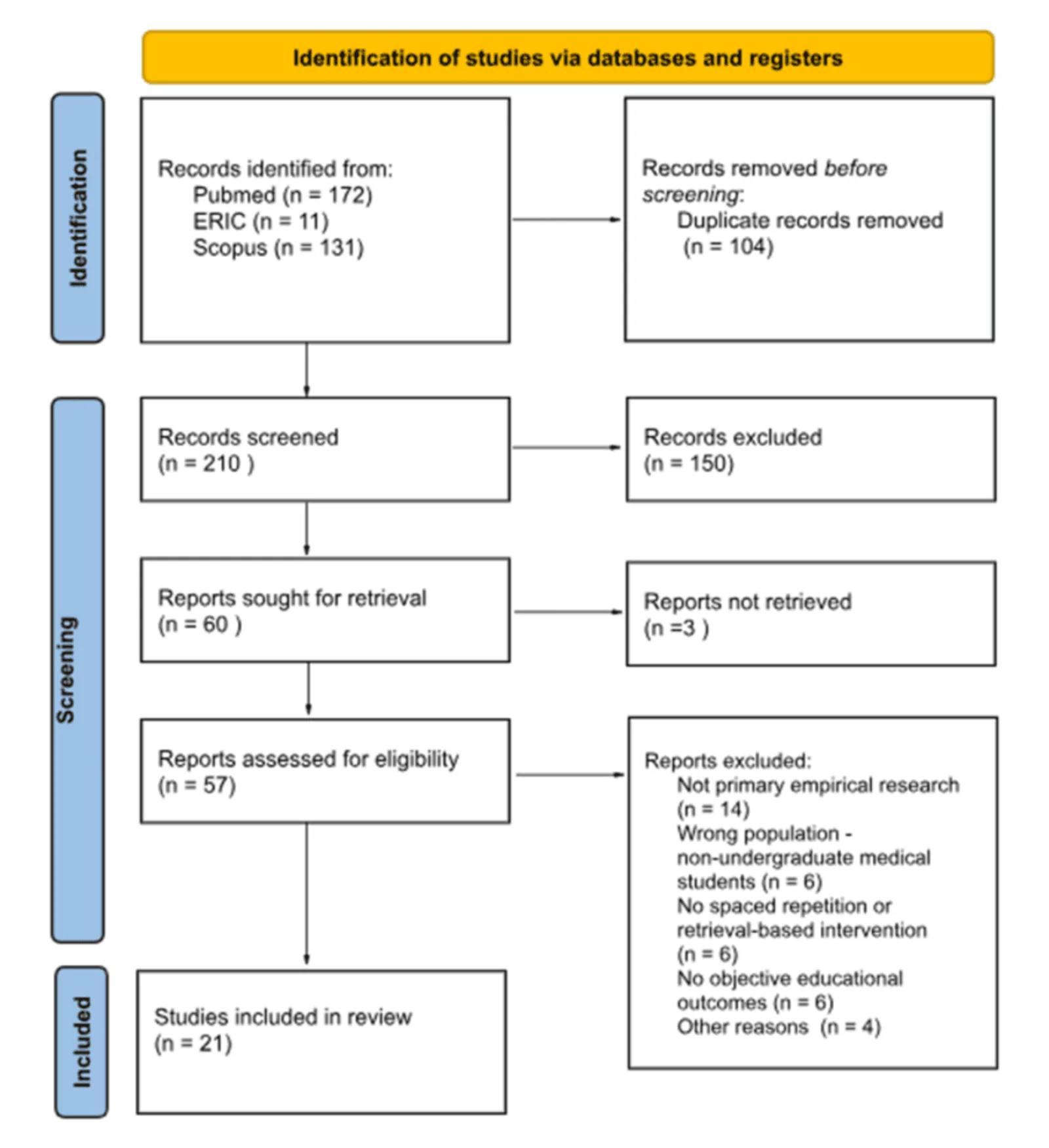
PRISMA flow diagram depicting the study selection This diagram illustrates the identification, screening, eligibility assessment, and inclusion of studies in this systematic review. A total of 314 records were identified through a search of the PubMed, ERIC, and Scopus databases. After removal of 104 duplicate records, 210 records were screened, of which 150 were excluded. Sixty reports were sought for retrieval, with three reports not retrieved. Fifty-seven studies underwent full-text review, and 21 studies met inclusion criteria. Reasons for exclusion included lack of primary empirical data, incorrect study population, absence of a spaced repetition or retrieval-based intervention, and lack of objective educational outcomes PRISMA: Preferred Reporting Items for Systematic Reviews and Meta-Analyses

Data Extraction

Variables that were extracted included study design, country, sample size, learner level, intervention modality, comparator type, duration of implementation, outcome measures, and direction of effect. Data were entered into a predefined extraction table for each study. A formally validated or pilot-tested extraction form was not used.

Outcome Classification

Outcomes were distinctly classified as either examination performance or knowledge retention. Examination performance included course and block examination scores, post-intervention assessment scores, NBME or Comprehensive Basic Science Examination (CBSE) scores, and United States Medical Licensing Examination (USMLE) scores. These outcomes were not considered direct measures of retention unless the assessment was administered after a defined interval following initial learning or completion of the intervention. Retention outcomes were limited to delayed assessments or repeated recall measurements conducted across prespecified intervals. Subjective factors such as perceived effectiveness, learner preference, and intervention usage alone were not considered objective academic outcomes.

Narrative Synthesis

A meta-analysis was not undertaken. The findings were synthesized narratively, and studies were grouped by intervention category and, within each category, by study design. This approach allowed structured curricular interventions and self-directed flashcard use to be compared while being reported separately. Within each group, the findings were summarized by the direction of effect on objective academic outcomes, with effect estimates reported as given in the source publications. The direction of effect was assigned exclusively based on objective academic outcomes. It is important to note that learner perceptions, preferences, and satisfaction did not contribute to the direction of effect. Conclusions regarding overall effectiveness were not drawn from counts of statistically significant results alone, and no pooled estimates or heterogeneity statistics were calculated. The rationale for not pooling is set out in the Discussion, and certainty of the evidence is addressed in a separate subsection.

Risk of Bias Assessment

Risk of bias was assessed using tools appropriate to each study design. Studies reporting random allocation were assessed with the Cochrane Risk of Bias 2 (RoB 2) tool across five domains: the randomization process, deviations from intended interventions, missing outcome data, measurement of the outcome, and selection of the reported result [29]. Each trial received an overall judgment of low risk of bias, some concerns, or high risk of bias. One trial described random allocation without reporting a sequence generation method, allocation concealment, or balanced group sizes; consistent with current guidance, it was retained as a randomized trial and judged accordingly within the randomization domain rather than reassigned to a non-randomized tool.

Non-randomized studies were assessed with the Risk Of Bias In Non-randomized Studies of Interventions (ROBINS-I) tool across seven domains: confounding, selection of participants into the study, classification of interventions, deviations from intended interventions, missing data, measurement of outcomes, and selection of the reported result [30]. Overall judgments were categorized as low, moderate, serious, or critical risk of bias, or no information. For observational studies of self-directed flashcard use, the intervention was defined as learner-initiated engagement with the platform. Because this exposure was not assigned, judgments within the confounding domain reflect the absence of allocation control and should be interpreted with that framing in mind. One study analyzed examination items rather than individual learners; ROBINS-I domains were applied to that study with the unit of analysis noted explicitly.

One reviewer (NKL) performed the initial assessments under the supervision of the senior author (SS). Assessments were not conducted independently in parallel. Uncertain judgments were discussed, reviewed against tool-specific guidance, and resolved by consensus.

Results

Study Characteristics

The 21 studies that met inclusion criteria were conducted in North America, Europe, South America, and South Asia [4-10,12-25]. Sample sizes ranged from 29 learners in a log-based observational study to 684 learners in a multi-year historical cohort [9,16]. One retrospective analysis evaluated 3,734 questions across 51 examinations rather than individual learners [21]. Six studies used randomized or non-randomized controlled designs [15,16,22-25]. The remaining fifteen were observational cohorts or cross-sectional analyses [4-10,12-14,17-21]. The majority of studies focused on preclinical learners, with the exception of four examining clinical-year cohorts [15,22-24].

Table 3 reports the key characteristics, including study design, participant population, and intervention format, as well as the primary outcome and direction of intervention effect of the included studies. Effect direction in Table 3 was assigned according to the reported result of the intervention or study strategy on objective academic outcomes [4-10,12-25]. Studies were categorized as positive when examination performance or retention improved, neutral when no difference was found, mixed when there was only benefit on some outcomes, subgroups, or study arms but not others, and negative when outcomes favored the comparator [4-10,12-25]. Across the 21 included studies, effect direction was categorized as positive in 10 studies, mixed in five, and neutral in five. No study reported a negative effect of a retrieval-based strategy. The negative association reported by Clemmons et al. was for educational video use, the passive comparator, rather than for retrieval practice [4-10,12-25].

**Table 3:** Summary of included studies and direction of effect Effect direction was categorized based on the reported direction of association or intervention effect on objective academic outcomes. Positive indicated improved examination performance, knowledge retention, or other objective academic outcomes. Neutral indicated no difference in outcome. Mixed indicated benefit on some outcomes, subgroups, or study arms but not others. Coding was based exclusively on objective academic outcomes; learner perceptions, preferences, and satisfaction did not contribute to effect-direction assignment. Study designs were categorized as randomized controlled trials, controlled cohort studies (including both prospective and historical comparisons with a defined comparator group), and observational studies without a defined control group. Higher-intensity use refers to greater engagement with the intervention (e.g., increased number of flashcards reviewed, frequency of retrieval sessions, or cumulative exposure over time). Preclinical years refer to the initial phase of undergraduate medical education focused on foundational sciences, whereas clinical years refer to training involving patient-centered learning in clinical settings. Block examinations refer to course-specific assessments administered at the end of a defined instructional period RCT: randomized controlled trial; ARS: audience response system; MCQ: multiple-choice question; NBME: National Board of Medical Examiners; CBSE: Comprehensive Basic Science Examination (a standardized exam assessing foundational medical knowledge); VP: virtual patient; SAQ: short-answer question

Study	Design	Participants (n), population	Intervention format	Primary outcome	Main findings	Reported effect estimate	Effect direction
Curriculum-embedded retrieval quizzing							
Cunningham et al., 2024 [15]	Quasi-experimental (site-allocated)	140, clinical-year	Longitudinal spaced ECG quizzes	Post-test score	Higher post-test scores than both control groups	Hedges' g = 1.53 vs LIC control; g = 0.90 vs block control	Positive
Venkatesh et al., 2024 [17]	Analytical cross-sectional	150, preclinical	Formative retrieval ("keyword recall")	Summative assessment performance	Keyword-recall formative scores correlated with summative performance more strongly than MCQ formative scores; no comparator group	r = 0.28 (MCQ); r = 0.35 (keyword recall)	Mixed
Donker et al., 2022 [21]	Retrospective item-level analysis	3,734 questions across 51 examinations, preclinical	Longitudinal spaced retrieval questions	Performance across repeated exposure	Knowledge did not significantly decline across repeated retrieval opportunities	NR	Neutral
Vagha et al., 2025 [24]	Quasi-experimental (sequential allocation)	90, clinical-year	Structured spacing intervals	Post-test MCQ score	Baseline scores comparable (11.42 vs 11.58); post-test significantly higher in intervention group (16.24 vs 11.89, p < 0.0001)	NR	Positive
Audience response system retrieval in live teaching							
Anders et al., 2022 [16]	Historical cohort	684, preclinical	ARS-based retrieval integration	NBME performance	Associated with higher NBME performance than in prior cohorts	NR; 95% CI displayed graphically only	Positive
Faculty created flashcards with a structured schedule							
Santos-Ferreira et al., 2024 [20]	Prospective cohort	558 exploratory, 269 confirmatory; preclinical	Moodle Leitner-system flashcards	Physiology exam score	Higher flashcard volume showed an apparent dose-response association with physiology exam performance	β per 100 cards reviewed: 0.75 (95% CI 0.24-1.26); 0.44 (0.16-0.71); 0.56 (0.31-0.81)	Positive
Durrani et al., 2024 [23]	Randomized (method not reported)	115, clinical-year	Faculty-created Anki flashcards	MCQ exam score	Intervention scores rose from 27.93 to 30.80; control group showed no significant improvement	Effect size 0.8; 95% CI NR	Positive
Faculty provided flashcards; learner-scheduled use							
Magro et al., 2024 [18]	Retrospective QI cohort	104 intervention, 102 control; preclinical	Faculty-generated Anki decks	Course exam performance	No significant difference in pharmacology exam scores; students reported positive perceptions	NR	Neutral
Sun et al., 2021 [19]	Prospective cohort	101, preclinical	Psychiatry flashcard deck	Course exam score	Flashcard use was well received but not associated with higher psychiatry exam scores	NR	Neutral
Comparative retrieval formats							
Gianotto-Oliveira et al., 2026 [25]	Stratified RCT	80, preclinical	Virtual patient, MCQ, or SAQ retrieval practice	Retention test performance	Short-answer retrieval produced significant improvement; other arms maintained retention without significant gain	Estimated marginal means with 95% CI (repeated SAQ, MCQ group 0.665, 95% CI 0.602-0.728)	Mixed
Platform-based study plus quiz with self-graded recall							
Taveira-Gomes et al., 2015 [22]	RCT	96, clinical-year	Spaced study plus retrieval sessions	Self-graded recall accuracy†	Study-plus-quiz group achieved greater gains in recall accuracy than the quiz-only group across sessions	NR	Positive
Practice-question bank use							
Clemmons et al., 2023 [5]	Retrospective cohort	327, preclinical	Practice questions versus educational videos	USMLE Step 1 score	Practice question use positively predicted Step 1 scores; video use showed a negative association	β = 0.141 (2022) and β = 0.133 (2023) for practice questions; β = −0.118 for videos (2023)	Mixed
Self-directed spaced repetition software, learner-scheduled							
Haughey et al., 2025 [4]	Prospective cohort	43 matched pre/post, preclinical	Anki intensity	Percentage increase in test score	No overall benefit for Anki use; higher-intensity users and physiology-specific use showed greater score improvement	NR	Mixed
Deng et al., 2015 [6]	Retrospective survey cohort	72, preclinical	Self-directed Anki	USMLE Step 1 score	Unique flashcards reviewed was an independent predictor of Step 1 score	Standardized β 0.195 (95% CI 0.027-0.363)	Positive
Gilbert et al., 2023 [7]	Cohort-control	130, preclinical	Self-directed Anki	Course exams and CBSE	Anki users scored higher than non-users on all four assessments after adjustment for MCAT percentile	MCAT-adjusted mean differences 6.2 (95% CI 2.4-9.9) to 10.7 (95% CI 2.6-18.7)	Positive
Lu et al., 2021 [8]	Retrospective survey cohort	201, preclinical	Self-directed Anki	USMLE Step 1 score	Meaningful Anki users scored higher than non-users (241.1 ± 13.2 vs 235.5 ± 17.7; p = 0.012)	NR	Positive
Mehta et al., 2023 [9]	Retrospective cohort	29, preclinical	Log-based Anki use	Summative NBME performance	Students above the class median had studied more total flashcards and more flashcards per day than those below	NR	Positive
Winter et al., 2025 [10]	Observational cohort	36, preclinical	Mature cards, study hours, unsuspended cards	CBSE score	Students with above-average mature card counts scored higher than those below the mean (71.5% vs 60.0%)	NR	Positive
Spence et al., 2026 [12]	Cross-sectional	176, preclinical	Self-directed Anki	Written and laboratory exam performance	No performance advantage with Anki; wellness and sleep correlated more strongly with written exam scores	Correlation coefficients only; no CI	Neutral
Wothe et al., 2023 [13]	Cross-sectional survey	165, preclinical	Daily Anki use	USMLE Step 1 and Step 2 scores	Daily Anki use was associated with higher Step 1 scores, but no comparable association was seen for Step 2	NR	Mixed
Levy et al., 2023 [14]	Prospective cohort	45, preclinical	Flashcard use intensity	Block exam score	Intermediate and heavy users had numerically higher scores, but the difference did not reach statistical significance	NR for examination outcomes; 95% CI reported for survey items only	Neutral

The included studies evaluated several related, but non-equivalent, constructs. Spaced repetition refers to distributing exposure to material across time, whereas retrieval practice refers to actively recalling material rather than restudying it. Flashcard exposure and practice question use are specific implementations that may involve one or the other or both. Curriculum-embedded testing refers to institutionally scheduled retrieval opportunities delivered independently of learner initiative. Most self-directed flashcard studies combined spaced repetition and retrieval practice without separating their contributions, and none was designed to isolate the effect of either component. These constructs are therefore reported separately in Table 3 and should not be treated as interchangeable.

Intervention Characteristics

Spaced repetition and retrieval-based practices were delivered through distinct formats across studies. The most common modality was Anki flashcard use, which was typically studied in observational analyses of preclinical students who independently adopted the platform as part of their study routine and were self-directed [4,6-10]. In these studies, students used cards made and shared by other students, self-made cards, or a combination of both. Anki exposure was quantified through student survey responses, usage logs, or card review statistics. The Anki-based interventions are not as standardized as curriculum-based approaches because the students who used Anki determined their own learning schedule, timing, card volume, and pattern of use.

A second group of studies evaluated faculty-developed or curriculum-embedded spaced repetition systems [15-18,20-22,24] and were often delivered through Moodle or similar digital learning platforms [18,20]. These interventions were more structured than self-directed flashcard or Anki use and often incorporated flashcard sets made by instructors, course-specific content, or standardized review schedules. Repetition was built intentionally into the learning design by educators rather than being left for learners to implement independently. Other interventions in this group of studies utilized retrieval quizzes embedded in the curriculum that were sometimes delivered longitudinally over weeks or months and sought to reinforce previously taught material [15,17,21,22,24]. Some investigators used repeated multiple-choice quizzes or short-answer formats, while others incorporated retrieval into live teaching sessions through audience response systems or classroom polling [16]. Together, the spaced repetition practices in these studies were not of a single implementation type but were a spectrum of approaches that varied in both structure and learner autonomy. Table 4 elaborates on the most frequently recurring study methods found in the included studies.

**Table 4:** Most common forms of spaced repetition interventions encountered The most frequently recurring study methods identified in the included studies and a description of their structure, learner participation habits, duration, intensity measures, and overall outcome patterns. Categories were assigned according to shared implementation features. The typical outcome pattern reflects the general direction of findings reported within each category rather than the results of any single study

Intervention category	Typical format	Learner participation	Typical duration	Common intensity measure	Typical outcome pattern
Self-directed flashcard use	Independent use of Anki or similar digital flashcard platforms, usually with student-selected pre-made or self-created decks	Voluntary/self-selected	Variable; typically weeks to months, sometimes for an entire preclinical year	Total cards reviewed, mature cards, study hours, days of use, consistency of review	Modest-to-moderate positive associations; more consistent and higher-volume use was associated with better performance
Curriculum-embedded flashcards	Faculty-developed flashcards or institutionally provided digital decks integrated into course content through Moodle or similar platforms	Usually required or institutionally provided; actual use varied	Single module, semester, or academic year	Number of assigned decks completed, session frequency, cumulative exposure across the course	More structured effects; benefits were more apparent when flashcards were repeatedly revisited over time
Structured retrieval quizzes	Repeated quizzes delivered at planned intervals after initial instruction, including online quizzes, spaced classroom tests, or repeated post-session assessments	Usually required as part of the study design or course activity	Multiple weeks to several months; sometimes repeated across an academic term	Number of quiz sessions, frequency of quiz exposure, delayed test performance	Moderate-to-large positive effects, especially when repeated over multiple time points
Lecture-integrated retrieval	Retrieval prompts embedded within teaching sessions, such as audience response systems, polling, or in-class recall activities	Usually required during scheduled teaching sessions	Single session to repeated use across a course or clerkship block	Participation rate, number of retrieval prompts per session, intentional repetition across lectures	Positive improvement, particularly when used repeatedly across several sessions rather than in isolated lectures

The duration of interventions also varied. Some studies evaluated relatively short periods of time, such as a one-month or even a single-module intervention [19,23]. Longer studies spanned a semester or an academic year [15,20,21]. Some incorporated repeated examinations throughout a time period of several months [15,16,20,21,24]. Shorter interventions assessed immediate or near-term effects on course performance, while longer implementations evaluated whether long-term repeated retrieval influenced cumulative examination performance, NBME scores, or licensing-related outcomes [6,8,15,16]. This variation in duration is important because the theoretical basis of spaced repetition is that repeated exposure over time is a key mechanism by which forgetting is diminished, and retention is strengthened.

Intensity of exposure was measured differently across the studies. In learner-directed Anki studies, intensity was most commonly quantified by the number of flashcards reviewed, the number of mature cards, study hours, or consistency of daily use [4,6-10,13]. In curriculum-based studies, intensity was reflected by the number of scheduled quizzes, frequency of retrieval sessions, or cumulative exposure to repeated assessment opportunities [15,16,20,21,24]. The recurring pattern in these studies was that interventions with greater repetition, longer duration, or more consistent participation were more likely to demonstrate measurable academic benefit.

Across studies, reviewing over longer periods of time and engaging at higher levels of intensity were consistently associated with improved academic outcomes [15,20,21,24]. Interventions that incorporated repeated retrieval over extended periods tended to demonstrate stronger effects than shorter or lower-intensity implementations [4,6-10,12-14]. This pattern is consistent with an apparent dose-response association, though intensity of engagement was not randomly assigned in any included study. At the same time, the heterogeneity of intervention designs means that no single implementation model can yet be identified as clearly superior. Rather, the available evidence suggests that both structure and sustained engagement are important features of effective spaced repetition interventions in undergraduate medical education.

Controlled Interventions

Randomized and controlled studies reported improved examination performance and retention when learning sessions utilizing retrieval practice were spaced apart. For example, Taveira-Gomes et al. randomized 96 medical students to either a quiz-only group or a study-plus-quiz group. The quiz was delivered across three consecutive laboratory sessions (s0, s1, and s2), with each session separated by one week [22]. Students were not tested with a standard course examination. Instead, participants systematically studied material prescribed by learning objectives and then completed a quiz using open-ended prompts, after which recall accuracy was self-rated by comparing the user’s responses with the correct answers. In the first session (s0), both groups completed only the quiz; in the next two sessions (s1 and s2), the intervention group completed a task designed to help them study the material followed by the quiz, whereas students in the control group completed only the quiz. The study-plus-quiz group demonstrated greater gains in recall accuracy over time, which suggested that repeated retrieval, combined with repeated exposure to the material, improved retention [22]. Similarly, Cunningham et al. reported that implementation of spaced ECG quizzes across a clinical clerkship year resulted in significantly higher post-test scores compared with control groups receiving standard instruction [15].

Other quasi-experimental and controlled cohort studies also reported improvements in performance on course assessment. Durrani et al. studied 115 undergraduate medical students in pediatrics and found that a four-week, faculty-created Anki intervention produced significantly higher post-intervention scores on formative assessments compared with traditional book- and lecture-based study methods [23]. Mean scores of participants in the Anki group increased from 27.93 ± 4.53 to 30.80 ± 4.56. Compared to the scores of participants in the control group, which showed no significant improvement, the reported effect size was large (Cohen’s d = 0.8) [23].

In another controlled cohort study, Vagha et al. evaluated 90 final-year medical students in pediatrics and found that structured spaced-repetition significantly improved post-test performance compared to traditional study methods involving intensive concentrated study sessions of clinic notes and textbooks without periodic reinforcement [24]. Initial baseline pre-test scores were similar between groups (11.42 vs 11.58), but the intervention group achieved a significantly higher post-test score (16.24 vs 11.89; p < 0.0001) [24]. For each of the controlled interventions, improvements were most consistently observed when retrieval activities were repeated over multiple weeks or embedded within the curriculum across multiple teaching sessions, course blocks, or academic terms instead of as a one-time occurrence.

Observational Studies

Findings among observational studies were mixed. Several studies of voluntary flashcard use identified higher academic performance among students engaging in spaced repetition, while others found no association. One observational study found that increased use of educational videos, a passive learning strategy, was negatively associated with examination performance, while active retrieval through practice questions was associated with improved outcomes [5]. Studies measuring voluntary flashcard use identified higher academic performance among students engaging in spaced repetition. A cross-sectional survey of 72 medical students preparing for USMLE Step 1 conducted by Deng et al. found that the number of unique Anki flashcards reviewed during the preclinical period was an independent predictor of Step 1 score, with approximately one additional point gained for every 1,700 cards reviewed [6].

Similarly, Lu et al. studied 201 medical students and reported that students who identified as meaningful Anki users - identified by a survey consisting of multiple-choice questions, Likert-scale questions, and free-response questions - scored significantly higher on USMLE Step 1 than nonusers (241.1 ± 13.2 vs 235.5 ± 17.7; p = 0.012) [8]. This same study also found that students who completed reviews of previously studied decks consistently achieved the highest scores. This suggests that use, frequency, and completeness of review all influence performance [8]. Additional observational studies examining course-level outcomes reported similar patterns. Gilbert et al. found that Anki users scored significantly higher than non-users on all four assessments after adjustment for MCAT percentile [7]. Mehta et al. grouped first-year medical students by performance on summative NBME examinations and found that students above the class median had studied more total flashcards, and more flashcards per day, than those below [9]. However, effect sizes in these observational analyses were smaller than those of controlled interventions. In addition, the conclusions and generalizability were limited by potential self-selection and motivation bias.

Apparent Dose-Response Patterns

Several studies across different designs suggested an apparent dose-response association between retrieval practice intensity and academic outcomes. Santos-Ferreira et al. identified a “dose-dependent” association between the number of digital flashcards reviewed and physiology examination performance. In this study, there was a statistically significant positive correlation between the number of flashcards studied and all three exam grades [20]. Haughey et al. stratified first-year medical students by total Anki review activity - defined by the number of cards reviewed and recorded in the Anki app - and found that students with higher review volumes demonstrated greater improvements between pre- and post-module test scores than lower-intensity users [4].

Other studies reported similar trends, suggesting that both the timing and consistency of spaced repetition use may positively affect academic outcomes [10,13,14]. Winter et al. found that medical students with above-average mature card counts, which is defined as a greater number of flashcards that had reached review intervals longer than 21 days, scored substantially higher on the CBSE than students who scored below the mean (71.5% vs 60.0%). CBSE scores were also positively correlated with total study hours, number of mature cards, and number of cards being actively reviewed [10]. Wothe et al. similarly reported that students who used Anki daily achieved higher USMLE Step 1 scores than students who used Anki less frequently, although no comparable benefit was seen for Step 2 performance [13].

In contrast, Levy et al. grouped 45 first-year medical students in an eight-week anatomy and physiology course by the intensity of Anki use, which was determined according to the following metrics: the percentage of study days on which Anki was used, the number of flashcards reviewed per day, and the average time spent per card. Heavy and intermediate users were defined as students who used Anki at least 90% or 75% of course days, respectively, while meeting minimum thresholds of 75 flashcards per day and five seconds per card. These groups achieved higher scores on course examinations written by faculty than light or limited users (90.34% and 91.74% vs 85.86% and 87.75%, respectively), although the overall difference did not reach statistical significance [14].

Together, these findings describe an apparent dose-response association between cumulative retrieval activity and examination performance. Because intensity of use was learner-determined in every study reporting this pattern, greater flashcard volume, higher mature card counts, and more frequent daily use may reflect underlying motivation or baseline academic strength rather than an effect of exposure itself. Levy et al. reported numerically higher scores among heavier users that did not reach statistical significance, which is consistent with this interpretation.

Considered separately, the two bodies of evidence differ in both strength and consistency. Among studies with a defined comparator, benefit on objectively scored assessments was reported consistently, though no such study was judged at low risk of bias and sample sizes were small. Among observational studies of self-directed flashcard use, findings were mixed: several reported positive associations with course or licensing examination performance, while others, including studies that measured engagement objectively from application logs, found none. Because adoption was learner-dependent in every observational study, these associations cannot be separated from differences in motivation, baseline academic ability, prior knowledge, established study habits, or access to shared decks and commercial resources.

Risk of Bias

No included study was determined to be at low risk of bias. Of the three studies reporting random allocation, two were rated as raising some concerns and one as high risk of bias. The high-risk study was because randomization was asserted without a described method and with unequal group sizes. Among the two trials rated as raising some concerns, the recurring limitations were unreported allocation concealment, the absence of participant blinding inherent to educational interventions, and the absence of a registered protocol against which reported outcomes could be compared.

Of the 18 non-randomized studies, four were judged at moderate and 14 at serious risk of bias. Confounding was the domain that most often drove the overall decision. Studies of self-directed flashcard use compared learners who chose to adopt the platform with those who did not, and most did not adjust for baseline academic performance; where baseline characteristics were compared, users differed systematically from non-users. The four studies rated moderate shared three features: exposure measured from platform logs or registrar records rather than self-report, outcomes drawn from official examination results, and regression models incorporating admission or prior academic performance. Selection into the study was a second recurring concern, with survey response rates in the observational set ranging from 18.6% to 65%. Measurement of outcomes was the strongest domain overall, since most studies used course examinations, national board examinations, or licensing examination scores obtained from institutional records.

The domain classifications are presented in Tables 5-6, and the distribution of overall decisions is presented in Table 7.

**Table 5:** RoB 2 domain judgments for randomized trials Domains are those of the RoB 2 tool: D1: randomization process; D2: deviations from intended interventions; D3: missing outcome data; D4: measurement of the outcome; D5: selection of the reported result. Overall judgments follow RoB 2 algorithm conventions, in which a trial is rated as having some concerns when at least one domain raises some concerns, and no domain is at high risk RoB 2: Cochrane Risk of Bias 2

Study	D1 Randomization	D2 Deviations	D3 Missing data	D4 Outcome measurement	D5 Reported result	Overall
Taveira-Gomes et al., 2015 [22]	Some concerns	Some concerns	Low	Some concerns	Some concerns	Some concerns
Gianotto-Oliveira et al., 2026 [25]	Some concerns	Some concerns	Some concerns	Low	Some concerns	Some concerns
Durrani et al., 2024 [23]	High	Some concerns	Some concerns	Some concerns	Some concerns	High

**Table 6:** ROBINS-I domain judgments for non-randomized studies This table depicts domains of the ROBINS-I tool: confounding; selection of participants into the study; classification of interventions; deviations from intended interventions; missing data; measurement of outcomes; and selection of the reported result. For studies of self-directed flashcard use, the intervention was defined as learner-initiated engagement with the platform ROBINS-I: Risk Of Bias In Non-randomized Studies of Interventions; L: low; M: moderate; S: serious; C: critical

Study	Design	Confounding	Selection	Classification of intervention	Deviations	Missing data	Outcome measurement	Reported result	Overall
Clemmons et al., 2023 [5]	Retrospective cohort	M	L	L	M	L	L	M	Moderate
Santos-Ferreira et al., 2024 [20]	Prospective cohort	M	M	L	M	M	L	M	Moderate
Sun et al., 2021 [19]	Prospective cohort	M	M	M	M	M	L	M	Moderate
Vagha et al., 2025 [24]	Sequentially allocated controlled	M	M	L	M	L	M	M	Moderate
Anders et al., 2022 [16]	Historical cohort	S	M	M	S	M	M	M	Serious
Cunningham et al., 2024 [15]	Site-allocated controlled	S	M	L	M	M	M	M	Serious
Deng et al., 2015 [6]	Retrospective survey cohort	M	S	S	M	M	L	M	Serious
Donker et al., 2022 [21]	Retrospective item-level analysis	S	M	L	M	S	M	M	Serious
Gilbert et al., 2023 [7]	Cohort-control	S	M	M	M	M	L	M	Serious
Haughey et al., 2025 [4]	Prospective cohort	S	S	M	M	M	M	M	Serious
Levy et al., 2023 [14]	Prospective cohort	S	M	S	M	M	M	M	Serious
Lu et al., 2021 [8]	Retrospective survey cohort	S	S	S	M	S	L	M	Serious
Magro et al., 2024 [18]	Retrospective QI cohort	S	M	S	S	S	M	M	Serious
Mehta et al., 2023 [9]	Retrospective cohort	S	S	L	M	S	L	M	Serious
Spence et al., 2026 [12]	Cross-sectional	S	M	S	M	M	M	M	Serious
Venkatesh et al., 2024 [17]	Analytical cross-sectional	S	S	M	M	M	M	M	Serious
Winter et al., 2025 [10]	Observational cohort	S	S	L	M	M	L	M	Serious
Wothe et al., 2023 [13]	Cross-sectional survey	S	S	S	M	S	L	M	Serious

**Table 7:** Overall distribution of risk-of-bias judgments The judgments are reported separately by tool because RoB 2 and ROBINS-I generate non-equivalent categories and should not be pooled into a single scale. Of the 21 included studies, three were assessed with RoB 2 and 18 with ROBINS-I. No study was judged to be at low risk of bias RoB 2: Cochrane Risk of Bias 2; ROBINS-I: Risk Of Bias In Non-randomized Studies of Interventions

Tool	Judgment	Number of studies
ROB 2 (n = 3)	Low risk of bias	0
	Some concerns	2
	High risk of bias	1
ROBINS-I (n = 18)	Low	0
	Moderate	4
	Serious	14
	Critical	0

Discussion

This systematic review synthesizes evidence on spaced repetition and retrieval practice in undergraduate medical education, restricted to objective academic outcomes and covering both structured curricular interventions and self-directed flashcard use. It suggests that spaced repetition and retrieval practice are associated with improved academic outcomes in undergraduate medical education. The most consistent evidence of benefit came from randomized and controlled interventions, although none of these was judged to be at low risk of bias, and the most consistent benefit was observed where retrieval was structured and sustained [15,22-25]. Across the included literature, there were more consistent associations with academic performance for repeated, spaced engagement than for isolated or short-term review, although the strength of this inference varied considerably by study design. It is important to note that the magnitude of benefit was not uniform across all studies.

Importantly, these findings should be interpreted within the context of the broader educational literature. The trends in recent systematic reviews from the field of medical education suggest that spaced repetition is effective across a wide range of learner groups and training settings; however, the optimal scheduling and design of such interventions remain uncertain [1,2,11]. In addition, the findings of this review reinforce the concept that active retrieval practices are more beneficial than passive learning methods. This current review extends the literature by focusing specifically on undergraduate medical students and by synthesizing not only controlled interventions but also the rapidly growing body of observational studies examining self-directed Anki use. In this sense, the present review contributes a more focused understanding of how spaced repetition and retrieval practice function in the undergraduate medical setting, where students face a particularly large information load and frequent high-stakes testing.

A key pattern that emerged from the included literature was the difference between structured interventions and self-directed usage studies. Controlled studies typically evaluated defined educational strategies such as spaced quizzes, scheduled retrieval sessions, or curriculum-embedded flashcard systems delivered over multiple weeks or months [15-17,22-25]. In these studies, the timing and format of retrieval were specified in advance, and the outcomes were usually measured against a comparator condition. In contrast, observational studies most often examined voluntary student use of Anki or flashcard-based study methods [4-10,12-14]. These studies frequently reported positive associations with course examination performance, NBME examinations, or USMLE Step 1 scores. However, these studies were inherently more vulnerable to confounding. Students who choose to adopt highly structured retrieval systems may also be more motivated, more academically prepared, or more strategic in their study habits than students who do not. For this reason, the observational findings are best interpreted as supportive rather than definitive.

Another important theme was that the apparent benefit of spaced repetition was closely linked to duration and intensity of use. Several of the included studies suggested that retrieval practice was most efficacious when implemented repeatedly over time rather than as a brief or isolated intervention [15,20,21,24]. This was seen in both controlled and observational studies. For example, studies measuring flashcard volume, mature card counts, study hours, or daily use found that students with more consistent or higher-intensity engagement achieved stronger academic outcomes than lower-intensity users, although in one study this difference did not reach statistical significance [4,10,13,14,20]. These findings are consistent with the rationale for spaced repetition, which is based on repeated exposure and repeated retrieval across time instead of a single review session [1,3]. The available literature suggests that these methods are effective because students engage in repeated retrieval often and long enough to reinforce memory consolidation and not merely because learners are using flashcards or quizzes [1,3,21,22].

The results of this review are also notable because they span a range of educational settings and outcome types. Interventions in physiology, psychiatry, pharmacology, anatomy, pediatrics, and clinical clerkship instruction were studied [15-21,23-25]. Outcomes ranged from short-term, post-module tests to course examinations, NBME scores, and licensing examination performance [4-10,16]. This breadth strengthens the practical relevance of the findings, because it suggests that the educational value of spaced repetition and active retrieval may not be confined to one subject area or one type of assessment. However, it also introduces important heterogeneity.

The studies differed substantially in study method structure, duration, learner autonomy, and intensity metrics; furthermore, they did not use a single common outcome measure. Quantitative pooling was not undertaken for several specific reasons. Outcomes were not commensurate, ranging from a self-graded judgment of learning, to post-module tests written by course faculty, to institutional course examinations, to NBME subject examinations, to USMLE Step 1 and Step 2 scores, with no outcome common to more than a minority of studies. Timing of outcome assessment ranged from immediately following a single laboratory session to licensing examinations taken more than a year after the exposure period. Effect measures were reported inconsistently as well: Hedges' g, Cohen's d, unstandardized and standardized regression coefficients, and Pearson correlation coefficients each appeared, and 12 of the 21 studies reported neither an effect size nor a confidence interval for their primary academic outcome.

Furthermore, comparators differed in kind, including no intervention, an alternative active strategy, a historical cohort, and in several observational studies, no comparator at all. Interventions spanned learner-scheduled flashcard use through faculty-scheduled curriculum-embedded quizzing. Subgroup synthesis restricted to studies with a defined comparator was considered; however, it was not performed, since only three studies reported random allocation and the remainder were judged at moderate or serious risk of bias, so a pooled estimate would have carried a false impression of precision.

Practical Implications for Medical Schools and Curricular Design

Although the included studies varied in design and study method format, the overall pattern of findings is that undergraduate medical curricula may benefit from incorporating retrieval opportunities rather than leaving retrieval practice entirely to student preference. In both controlled and observational studies, repeated retrieval over multiple weeks, course blocks, or academic terms was more often associated with improved academic outcomes than isolated or short-term review activities [15-17,20-25]. This suggests that institutions may support learning most successfully by creating curricular structures that revisit prior material in a deliberate and recurrent manner. Examples include cumulative quizzes, repeated low-stakes assessments, spaced review sessions built into courses, and institutionally supported flashcard resources aligned with course objectives [15-18,20,21]. Although the available evidence does not identify a single superior implementation model, embedding retrieval across time may be more beneficial than relying on only end-of-course review.

Practical Implications for Faculty and Course Directors

Retrieval practice may be most useful for students when it is incorporated by faculty into the learning process as opposed to being used only as a testing strategy. In the studies included in this review, stronger effects were observed when learners were asked to retrieve previously taught material repeatedly over time instead of encountering it in only one intervention [15-17,20-25]. This has practical implications for course design. Faculty may consider using spaced quizzes after lectures, cumulative assessments that revisit earlier content, repeated in-class polling or audience response questions, and faculty-developed flashcard decks that reinforce foundational concepts across time [15-18]. Importantly, these strategies do not require a fully standardized or technology-heavy curriculum to be useful. Even relatively simple retrieval opportunities, when repeated intentionally, may help students retain material, though the available evidence does not establish the size of this benefit. The broader implication is that retrieval can be used not only to measure learning, but also to strengthen it.

Practical Implications for Students

For students, the potential benefit of spaced repetition is tied less to the specific platform used and more to the consistency, duration, and intensity of engagement. The findings of several observational studies show that students who reviewed a larger number of cards, accumulated more mature cards, studied more consistently, answered more practice questions, or used active retrieval strategies daily tended to achieve better academic outcomes than lower-intensity users [4,6-10,13,14,20]. No included study evaluated massed pre-examination use as a comparator, so the relative advantage of sustained over concentrated use remains untested. Instead, the literature supports beginning retrieval early, revisiting previously learned material regularly, and maintaining repeated exposure across weeks to months [4,6-10,13,14,20,21]. Students can also benefit from focusing not only on new content but on continued review of material learned previously, since the underlying purpose of spaced repetition is to reduce forgetting over time rather than simply increase short-term familiarity before an exam [1,3,21]. While no single study strategy is likely to be optimal for every learner, the available evidence supports active and repeated retrieval as a reasonable, low-risk, evidence-supported study strategy.

Practical Implications for Future Research

Future studies would do well to place greater emphasis on standardized, reproducible intervention designs. In particular, randomized trials at multiple institutions may help determine whether structured spaced retrieval strategies offer consistent academic benefit in different curricula and student populations. It also remains unclear which aspects of implementation are most important, such as the spacing interval, the number of repetitions, the extent of faculty involvement, and how effectively the intervention is integrated into the curriculum. Follow-up beyond performance on single-course examinations could also show whether exposure to spaced retrieval early in a student’s preclinical years leads to sustained knowledge retention during clinical training and later stages of education.

Certainty of the Evidence

A formal GRADE assessment was not undertaken, as no pooled effect estimates were generated. A structured appraisal informed by GRADE domains supports the following summary. For structured, curriculum-embedded retrieval interventions compared with usual study, certainty is low. Randomized and controlled evidence is available, and the direction of effect is consistent, but no study was judged at low risk of bias, sample sizes were small, and few studies reported effect estimates with confidence intervals. For self-directed flashcard use and academic performance, certainty is very low. This evidence is entirely observational; 14 of 18 non-randomized studies were judged at serious risk of bias with confounding the driving domain, and findings were inconsistent, with several of the better designed studies reporting no association. These certainty decisions should be read alongside the evidence of publication bias in this literature noted above.

Limitations of the Review

This review has methodological limitations independent of the included literature. A protocol was not registered in PROSPERO, or an equivalent registry, so the review's methods were not publicly specified in advance, and selective outcome reporting at the review level cannot be excluded. Searches were limited to three databases and did not include Embase, Web of Science, or PsycINFO. Gray literature sources, including dissertations, conference abstracts, and trial registries, were not searched, so eligible unpublished or non-indexed work may have been missed. Eligibility was also restricted to English-language publications, which introduces the possibility of language bias, as relevant studies published in other languages were not captured. A formal reporting-bias assessment was not conducted, as funnel-plot and regression-based methods require a pooled analysis that was not undertaken.

However, a recent meta-analysis of spaced repetition in medical education examined this question directly and found funnel-plot asymmetry, with Egger's test supporting the presence of publication bias; trim-and-fill analysis reduced the pooled effect, although it remained statistically significant [1]. The literature synthesized here may therefore overrepresent positive findings. Finally, study selection, data extraction, and risk-of-bias assessment were primarily conducted by one reviewer and were not performed independently in duplicate, which may have increased the possibility of selection or extraction error. Although uncertain decisions and the narrative synthesis were reviewed with the senior author, this supervisory process does not provide the same protection against reviewer error as fully independent duplicate assessment.

Limitations of the Included Studies

Several limitations should be considered when interpreting the findings of this review. The included studies were heterogeneous with respect to design, intervention type, duration, and outcome measures, which limited direct comparison and precluded a formal meta-analysis. The interventions themselves combined spaced repetition, retrieval practice, flashcard exposure, practice-question use, and curriculum-embedded testing in varying proportions, and because few studies isolated a single construct, this review cannot determine which component drives the observed associations.

Risk of bias was a further constraint. No included study was judged to be at low risk of bias, and the majority of non-randomized studies were judged to be at serious risk. Many studies were observational and relied on voluntary learner engagement with flashcard platforms, introducing a meaningful risk of self-selection bias. Some analyses adjusted for prior academic performance and baseline characteristics; however, residual confounding remains likely, as self-selected platform use could not be separated from underlying academic motivation or ability. The conclusions of this review should be read against that constraint.

Another limitation is that there was not a consistent definition of knowledge retention across studies. Most included studies evaluated examination performance and inferred retention from course or licensing examination results measured at varying time points, whereas a smaller group used delayed post-tests or repeated recall measurements over defined intervals. Consequently, the evidence supporting improved examination performance was more extensive than the evidence supporting long-term knowledge retention. This limitation is compounded by the lack of long-term follow-up, as no included study assessed outcomes more than approximately one year after the intervention period. It is therefore difficult to determine whether the reported improvements in examination performance or retention persist over longer periods of time.

Generalizability is also a concern. Most included studies were conducted at single institutions, and institution-specific findings may not translate to other curricular structures or learner populations. Finally, reporting of effect magnitude and precision was inconsistent across the included literature. Twelve of the 21 studies reported neither an effect size nor a confidence interval for their primary academic outcome, which limited direct comparison of effect magnitude across studies and contributed to the decision to synthesize findings narratively.

## Conclusions

Spaced repetition and retrieval practice are associated with improved academic outcomes in undergraduate medical education, and this association is most consistent where retrieval is structured, repeated, and sustained across weeks to months rather than delivered as an isolated activity. The strength of the underlying evidence is limited. No included study was judged to be at low risk of bias, most non-randomized studies were judged to be at serious risk, and the observational literature on self-directed flashcard use is confounded by motivation, baseline academic ability, and study habits that cannot be separated from the decision to adopt these tools. The available evidence is therefore sufficient to support spaced retrieval practice as a reasonable, low-risk, evidence-supported study strategy, but not to establish a causal benefit across formats, and particularly not for self-directed flashcard use. Adequately powered randomized trials across multiple institutions, with prespecified outcomes and follow-up extending beyond a single course, are needed to determine which components of spaced retrieval produce benefit and how large that benefit is. These conclusions should be interpreted in light of the review's own constraints, including the absence of protocol registration, the exclusion of gray literature, and evidence of publication bias in this literature.

## Appendices

**Table 8:** PRISMA 2020 checklist Complete PRISMA 2020 checklist indicating where each item is reported in this review. Items marked as not applicable were not relevant to a narrative synthesis in which no meta-analysis, data conversion, or sensitivity analysis was undertaken PRISMA: Preferred Reporting Items for Systematic Reviews and Meta-Analyses

Section and Topic	Item #	Checklist item	Location where item is reported
TITLE			
Title	1	Identify the report as a systematic review	Title, p. 1
ABSTRACT			
Abstract	2	See the PRISMA 2020 for Abstracts checklist	Abstract, p. 1
INTRODUCTION			
Rationale	3	Describe the rationale for the review in the context of existing knowledge	Introduction and Background, pp. 1-4
Objectives	4	Provide an explicit statement of the objective(s) or question(s) the review addresses	Introduction and Background (review question), p. 2
METHODS			
Eligibility criteria	5	Specify the inclusion and exclusion criteria for the review and how studies were grouped for the syntheses	Methods - Eligibility Criteria and Table 1, pp. 4-5; Outcome Classification, p. 6; Narrative Synthesis, pp. 6-7; grouping shown in Table 3, pp. 8-9
Information sources	6	Specify all databases, registers, websites, organisations, reference lists and other sources searched or consulted to identify studies. Specify the date when each source was last searched or consulted	Methods - Information Sources and Search Strategy, p. 5; Table 2, p. 5
Search strategy	7	Present the full search strategies for all databases, registers and websites, including any filters and limits used	Table 2, p. 5
Selection process	8	Specify the methods used to decide whether a study met the inclusion criteria of the review, including how many reviewers screened each record and each report retrieved, whether they worked independently, and if applicable, details of automation tools used in the process	Methods - Reviewer Workflow, p. 5; Study Selection, pp. 5-6
Data collection process	9	Specify the methods used to collect data from reports, including how many reviewers collected data from each report, whether they worked independently, any processes for obtaining or confirming data from study investigators, and if applicable, details of automation tools used in the process	Methods - Data Extraction, p. 6; Reviewer Workflow, p. 5
Data items	10a	List and define all outcomes for which data were sought. Specify whether all results that were compatible with each outcome domain in each study were sought (e.g. for all measures, time points, analyses), and if not, the methods used to decide which results to collect	Methods - Outcome Classification, p. 6
	10b	List and define all other variables for which data were sought (e.g. participant and intervention characteristics, funding sources). Describe any assumptions made about any missing or unclear information	Methods - Data Extraction, p. 6
Study risk of bias assessment	11	Specify the methods used to assess risk of bias in the included studies, including details of the tool(s) used, how many reviewers assessed each study and whether they worked independently, and if applicable, details of automation tools used in the process	Methods - Risk of Bias Assessment, p. 7. RoB 2 applied to studies reporting random allocation; ROBINS-I applied to non-randomized studies; assessed by one reviewer under supervision, not independently in duplicate
Effect measures	12	Specify for each outcome the effect measure(s) (e.g. risk ratio, mean difference) used in the synthesis or presentation of results	No common effect measure was used, as no meta-analysis was performed. Effect estimates as reported by each study are presented in Table 3, pp. 8-9
Synthesis methods	13a	Describe the processes used to decide which studies were eligible for each synthesis (e.g. tabulating the study intervention characteristics and comparing against the planned groups for each synthesis (item #5))	Methods - Eligibility Criteria, p. 4; Data Extraction and Outcome Classification, p. 6; Narrative Synthesis, pp. 6-7; Results, pp. 7-15
	13b	Describe any methods required to prepare the data for presentation or synthesis, such as handling of missing summary statistics, or data conversions	Not applicable - no data conversions or imputation were performed
	13c	Describe any methods used to tabulate or visually display results of individual studies and syntheses	Methods - Narrative Synthesis, pp. 6-7; Figure 4, p. 6; Tables 3-7, pp. 8-15
	13d	Describe any methods used to synthesize results and provide a rationale for the choice(s). If meta-analysis was performed, describe the model(s), method(s) to identify the presence and extent of statistical heterogeneity, and software package(s) used	Methods - Narrative Synthesis, pp. 6-7; rationale for not pooling in Discussion, p. 16; Results, pp. 7-15
	13e	Describe any methods used to explore possible causes of heterogeneity among study results (e.g., subgroup analysis, meta-regression)	Results - Intervention Characteristics, pp. 9-10; Controlled Interventions, p. 11; Observational Studies and Apparent Dose-Response Patterns, p. 12. Grouping by intervention category and study design only; no statistical subgroup analysis or meta-regression was performed
	13f	Describe any sensitivity analyses conducted to assess robustness of the synthesized results	Not applicable - no sensitivity analyses were conducted
Reporting bias assessment	14	Describe any methods used to assess risk of bias due to missing results in a synthesis (arising from reporting biases)	Discussion - Limitations of the Review, p. 17. No formal reporting-bias assessment was conducted, as funnel-plot and regression-based methods require a pooled analysis; published funnel-plot and Egger's test evidence for this literature is discussed
Certainty assessment	15	Describe any methods used to assess certainty (or confidence) in the body of evidence for an outcome	Discussion - Certainty of the Evidence, p. 17. Formal GRADE assessment was not undertaken as no pooled estimates were generated; a structured GRADE-informed appraisal is reported
RESULTS			
Study selection	16a	Describe the results of the search and selection process, from the number of records identified in the search to the number of studies included in the review, ideally using a flow diagram	Methods - Study Selection, pp. 5-6; Figure 4, p. 6
	16b	Cite studies that might appear to meet the inclusion criteria, but which were excluded, and explain why they were excluded	Methods - Study Selection, pp. 5-6 (Mishall et al. and Laurent and Phillips were excluded on full-text reassessment for reporting learner preference, perceived effectiveness, or platform usage without an objective academic outcome); Figure 4, p. 6
Study characteristics	17	Cite each included study and present its characteristics	Results - Study Characteristics, p. 7; Table 3, pp. 8-9
Risk of bias in studies	18	Present assessments of risk of bias for each included study	Results - Risk of Bias, pp. 13-15; Table 5, p. 13; Table 6, p. 14; Table 7, p. 15
Results of individual studies	19	For all outcomes, present, for each study: (a) summary statistics for each group (where appropriate) and (b) an effect estimate and its precision (e.g., confidence/credible interval), ideally using structured tables or plots	Table 3 (including the Reported effect estimate column), pp. 8-9; Results - Controlled Interventions, p. 11; Observational Studies and Apparent Dose-Response Patterns, p. 12. Twelve of 21 studies reported neither an effect size nor a confidence interval for the primary academic outcome
Results of syntheses	20a	For each synthesis, briefly summarise the characteristics and risk of bias among contributing studies	Results - Study Characteristics, p. 7; Intervention Characteristics, pp. 9-10; Risk of Bias, pp. 13-15
	20b	Present results of all statistical syntheses conducted. If meta-analysis was done, present for each the summary estimate and its precision (e.g. confidence/credible interval) and measures of statistical heterogeneity. If comparing groups, describe the direction of the effect	No meta-analysis or pooled statistical synthesis was performed. Direction of effect for each study is reported in Table 3, pp. 8-9; narrative synthesis in Results, pp. 11-12
	20c	Present results of all investigations of possible causes of heterogeneity among study results	Results - Intervention Characteristics, pp. 9-10; Apparent Dose-Response Patterns, p. 12 (narrative exploration only)
	20d	Present results of all sensitivity analyses conducted to assess the robustness of the synthesized results	Not applicable - no sensitivity analyses were conducted
Reporting biases	21	Present assessments of risk of bias due to missing results (arising from reporting biases) for each synthesis assessed	Discussion - Limitations of the Review, p. 17. No synthesis-level reporting-bias assessment was performed; published evidence of publication bias in this literature is discussed
Certainty of evidence	22	Present assessments of certainty (or confidence) in the body of evidence for each outcome assessed	Discussion - Certainty of the Evidence, p. 17. Low certainty for structured curriculum-embedded retrieval interventions; very low certainty for self-directed flashcard use
DISCUSSION			
Discussion	23a	Provide a general interpretation of the results in the context of other evidence	Discussion, pp. 15-18
	23b	Discuss any limitations of the evidence included in the review	Discussion - Limitations of the Included Studies, pp. 17-18
	23c	Discuss any limitations of the review processes used	Discussion - Limitations of the Review, p. 17
	23d	Discuss implications of the results for practice, policy, and future research	Discussion - Practical Implications for Medical Schools and Curricular Design, Faculty and Course Directors, Students, and Future Research, pp. 16-17; Conclusions, p. 18
OTHER INFORMATION			
Registration and protocol	24a	Provide registration information for the review, including register name and registration number, or state that the review was not registered	Methods - Methodology, p. 4 (review not prospectively registered); Discussion - Limitations of the Review, p. 17
	24b	Indicate where the review protocol can be accessed, or state that a protocol was not prepared	Methods - Methodology, p. 4 (no separate review protocol was prepared)
	24c	Describe and explain any amendments to information provided at registration or in the protocol	Not applicable - no registered protocol
Support	25	Describe sources of financial or non-financial support for the review, and the role of the funders or sponsors in the review	Additional Information - Disclosures, p. 21
Competing interests	26	Declare any competing interests of review authors	Additional Information - Disclosures, p. 21
Availability of data, code and other materials	27	Report which of the following are publicly available and where they can be found: template data collection forms; data extracted from included studies; data used for all analyses; analytic code; any other materials used in the review	Extracted study-level data are presented in Tables 3-7, pp. 8-15. No template data collection form, analytic code, or separate dataset was generated; a formally validated or pilot-tested extraction form was not used
